# Using Raman spectroscopy for early detection of resistance-breaking strains of tomato spotted wilt orthotospovirus in tomatoes

**DOI:** 10.3389/fpls.2023.1283399

**Published:** 2024-01-03

**Authors:** Isaac D. Juárez, MacKenzi X. Steczkowski, Senthilraja Chinnaiah, Axell Rodriguez, Kiran R. Gadhave, Dmitry Kurouski

**Affiliations:** ^1^ Department of Biochemistry and Biophysics, Texas A&M University, College Station, Texas, TX, United States; ^2^ Department of Entomology, Texas A&M University, College Station, Texas, TX, United States; ^3^ Texas A&M AgriLife Research, Amarillo, Texas, TX, United States

**Keywords:** tomato spotted wilt orthotospovirus, Raman spectroscopy, high performance liquid chromatography, resistance breaking (RB) strains, early detection, orthotospovirus tomatomaculae

## Abstract

Tomato spotted wilt (TSW) disease caused by tomato spotted wilt orthotospovirus (TSWV, *Orthotospovirus tomatomaculae*) poses a significant threat to specialty and staple crops worldwide by causing over a billion dollars in crop losses annually. Current strategies for TSWV diagnosis heavily rely on nucleic acid or protein-based techniques which require significant technical expertise, and are invasive, time-consuming, and expensive, thereby catalyzing the search for better alternatives. In this study, we explored the potential of Raman spectroscopy (RS) in early detection of TSW in a non-invasive and non-destructive manner. Specifically, we investigated whether RS could be used to detect strain specific TSW symptoms associated with four TSWV strains infecting three differentially resistant tomato cultivars. In the acquired spectra, we observed notable reductions in the intensity of vibrational peaks associated with carotenoids. Using high-performance liquid chromatography (HPLC), we confirmed that TSWV caused a substantial decrease in the concentration of lutein that was detected by RS. Finally, we demonstrated that Partial Least Squares-Discriminant Analysis (PLS-DA) could be used to differentiate strain-specific TSW symptoms across all tested cultivars. These results demonstrate that RS can be a promising solution for early diagnosis of TSW, enabling timely disease intervention and thereby mitigating crop losses inflicted by TSWV.

## 1 Introduction

In 2020, over 180 million tons of tomatoes were produced globally, making this botanical fruit the most economically important vegetable worldwide (Food and Agriculture Organization of the United Nations, 1998). Although Mexico holds the title of the world’s largest exporter of tomatoes, with exports worth $2.57 billion, in 2021, the United States exported tomatoes valued at $224 million (OEC, 2021). These and other economic factors make tomatoes central to food security in the Americas.

Plant viruses cause massive crop losses valued at several billion dollars annually (Mumford et al., 2016). Among them, tomato spotted wilt orthotospovirus (TSWV, *Orthotospovirus tomatomaculae*) is one of the most devastating, infecting over 1000 plant species from 90 plant families, including potatoes, tomatoes, peppers, and tobacco (Riley et al., 2012). TSWV virions are spherical (80-110 nm diameter) with an outer membrane composed of lipoproteins and glycoproteins (Adkins et al., 1995; Kikkert et al., 1999). TSWV is efficiently transmitted by several species of thrips, predominantly by western flower thrips (WFT), *Frankliniella occidentalis* in a persistent propagative manner (Wan et al., 2020). Early larval instars of thrips acquire TSWV while feeding on infected plant cells. Upon acquisition, TSWV moves from the midgut to the primary salivary glands of the larva and replicates at both sites in the thrips vector. Subsequently, thrips remain infectious for the rest of their life cycle and transmit the virus as they feed on new plants (Rotenberg et al., 2015).

Management strategies for TSWV rely on using single gene resistant cultivars and applying toxic pesticides for thrips control. However, because of the concealed nature of the feeding of thrips, most pesticides are either ineffective or partially effective against thrips (Gao et al., 2012). Furthermore, their intensive applications lead to pesticide resistance development in thrips (Wan et al., 2021). For crop resistance, *Sw-5b-* and *Tsw*-mediated single gene resistance was deployed in commercial cultivars of tomato and pepper, respectively (Boiteux and de Ávila, 1994; Boiteux, 1995; Dianese et al., 2011; de Oliveira et al., 2018). However, such a resistance has exerted tremendous selection pressure on TSWV, which led to the emergence of resistance breaking (RB) strains worldwide (Aramburu and Martí, 2003; Margaria et al., 2004; Ciuffo et al., 2005; Sharman and Persley, 2006; Margaria et al., 2007; Zaccardelli et al., 2008; Fidan and Sari, 2019; Yoon et al., 2021; Almási et al., 2023). In the US, tomato-infecting RB strains capable of infecting an array of commercial tomato cultivars have been reported in California, North Carolina, and most recently in Texas by the Gadhave lab (Batuman et al., 2016; Chinnaiah et al., 2023b; Lahre et al., 2023). These strains are genetically distinct as they possess unique mutations in the TSWV movement protein (NSm). We used two sympatric strains: Tom-BL1 and Tom-BL2 originating from Bushland, TX and two allopatric strains: Tom-CA originating from California, and Tom-MX originating from Mexico. NSm sequences of all RB strains shared 94-99% nucleotide and 97-99% amino acid homology in pairwise comparisons with other TSWV isolates reported earlier. Interestingly, RB strains have been reported to selectively offer fitness benefits to WFT and facilitate their transmission better than a non-RB strain (Chinnaiah et al., 2023a). Generic symptoms of TSW include necrotic rings and spots on leaf, petiole, and stem; chlorosis and bronzing of leaves followed by stunting and partial wilting of plants.

The most widely used diagnostic methods for TSWV are polymerase chain reaction (PCR) or protein-based analyses (Roberts et al., 2000; Chinnaiah et al., 2022; Gao and Wu, 2022; Iturralde Martinez and Rosa, 2023). Although accurate, both analyses are laborious, time-consuming, invasive and require significant technical expertise. Furthermore, both methods require sample shipment to testing facilities, which increases direct costs of diagnostics. Raman spectroscopy (RS) is a valuable tool that can be used to detect and identify changes in plant biochemistry. RS is based-on measuring Raman scattering, a phenomenon dependent on a sample’s molecular composition and structure. Observed biochemical changes within a crop can then be used to diagnose infection caused by plant pathogens. Previous studies have shown RS’s ability to detect fungal pathogens within wheat, sorghum, and corn using handheld-spectrophotometers (Egging et al., 2018; Higgins et al., 2023). Additionally, Mandrile et al. showed that RS could be used to detect both tomato yellow leaf curl Sardinia virus (TYLCSV) and TSWV in tomato crops (Mandrile et al., 2019). Expanding upon this, we examined the extent to which RS could be used for the early detection of RB-TSWV in tomato crops. In our study, we inoculated three different tomato cultivars of varying resistance to TSWV with four RB-TSWV strains. Our results indicated that RS could be used to detect early TSWV infection and even predict strain specific differences in TSW symptoms in tomato leaves.

## 2 Materials and Methods

### 2.1 Tomato cultivation and virus inoculation

In this study, TSWV-resistant (cv. Celebrity), moderately resistant (cv. Supremo), and susceptible (cv. Hot-Ty) cultivars of tomato were grown in pots containing peat moss under greenhouse conditions. Four RB strains: Tom-CA, Tom-MX, Tom-BL1 and Tom-BL2 were mechanically inoculated onto five three-week-old tomato plants from each cultivar per strain using 0.1M sodium phosphate buffer. Further details on the source of RB-TSWV strains and methods are provided in Chinnaiah et al., 2023b. Inoculated plants with different RB-TSWV strains were maintained in insect-proof cages separately till symptom expression in the greenhouse at 25°C with a 12-hr photoperiod. TSW symptomatic plants were tested to confirm the presence of TSWV using PCR analysis previously published by our group (Gautam et al., 2022; Chinnaiah et al., 2023b). Briefly, a TaqMan probe-based qPCR assay targeting a 200-bp region in nucleoprotein (N) of the TSWV using TSWV-F: 5′-AGAGCATAATGAAGGTTATTAAGCAAAGTGA-3′ and TSWV-R: 5′-GCCTGACCCTGATCAAGCTATC-3′ primers and TaqMan probe: 5′-CAGTGGCTCCAATCCT-3′ was used for the TSWV detection. Results of qPCR analysis are shown in **Supplementary Figure S1**. Furthermore, symptomatic leaves from all three cultivars were collected on 25^th^ day post inoculation and subjected to Raman spectroscopy and HPLC analyses. Non-infected tomato plants were used as a control in all experiments.

### 2.2 Raman spectroscopy

Using a Resolve Agilent handheld spectrophotometer, we collected 30 surface scan spectra from TSW symptomatic systemic leaves (including non-symptomatic non-infected control) for each strain and cultivar combination, except for the Hot-Ty non-infected control, which were unsuitable to scan due to external factors. The laser emitted light at a wavelength of 830 nm. Acquisition time was 1 s and the laser power was 495 mW. The spectrophotometer automatically baselined the spectra. The spectra were then normalized at the 1440 cm^-1^ peak using MATLAB. This vibrational band originates from CH_2_ vibrations that are present in nearly all biological molecules. Therefore, spectral normalization on 1440 cm^-1^ becomes the least biased to compare changes in the intensities of other vibrational bands that can be used to access disease-induced changes in plant biochemistry.

MATLAB equipped with PLS_Toolbox(EigenvectorResearchInc.) was used to analyze the acquired spectra. The spectra utilized for training the PLS-DA model were preprocessed through area normalization and mean centering. The PLS-DA models were built using all spectra collected, and true prediction rates (accuracy) were determined by cross-validation. ANOVA with a significance level (α) of 0.05 was used for statistical comparison of peak height.

### 2.3 High-performance liquid chromatography

Carotenoids were extracted by homogenizing 150 milligrams of tomato leaves with a mortar and pestle. Next, 1.5 mL of chloroform:dichloromethane (2:1, v/v) was added to the mashed plant tissue; the mixture was agitated on a thermomixer at 4°C and at 500 rpm for 30 min. After that, 0.5 mL of 1 M sodium chloride was added to induce phase separation. The resultant solution was centrifuged at 7,000 rpm for 10 min and then phase separated. The organic phase was collected, and the aqueous phase underwent another round of phase separation after the addition of 0.75 mL chloroform:dichloromethane. The second round of organic phase was combined with the first organic phase collected and then dried using a Multivapor™ vacuum evaporator. Finally, the dried pellet was resuspended in 1mL 95% methanol prior to HPLC injection.

Plant extracts were analyzed using reverse-phase high-performance liquid chromatography (RP-HPLC). The HPLC instrument comprised a Waters 1525 pump in conjunction with the Waters 2707 autosampler and the 2489 Waters photodiode array detector. A C_30_ stationary phase, 3 μm particle size column with the dimensions 250 × 4.6 mm (Thermo Fisher Scientific Inc, part number 075723) was used for RP-HPLC. The mobile phases consisted of (A) a mixture of methanol and water (95:5, v/v) and (B) methyl tert-butyl ether (MTBE). The elution gradient was 97% A (0-6 min), followed by a linear decrease of A from 97% to 0% to 20 min. Lastly, the concentration of A was restored to 97% by 23 min. The detection of elution peaks was done at 450 nm. ANOVA with a significance level (α) of 0.05 was used for statistical comparison of area under the curve.

## 3 Results and discussion

**Figure 1:**
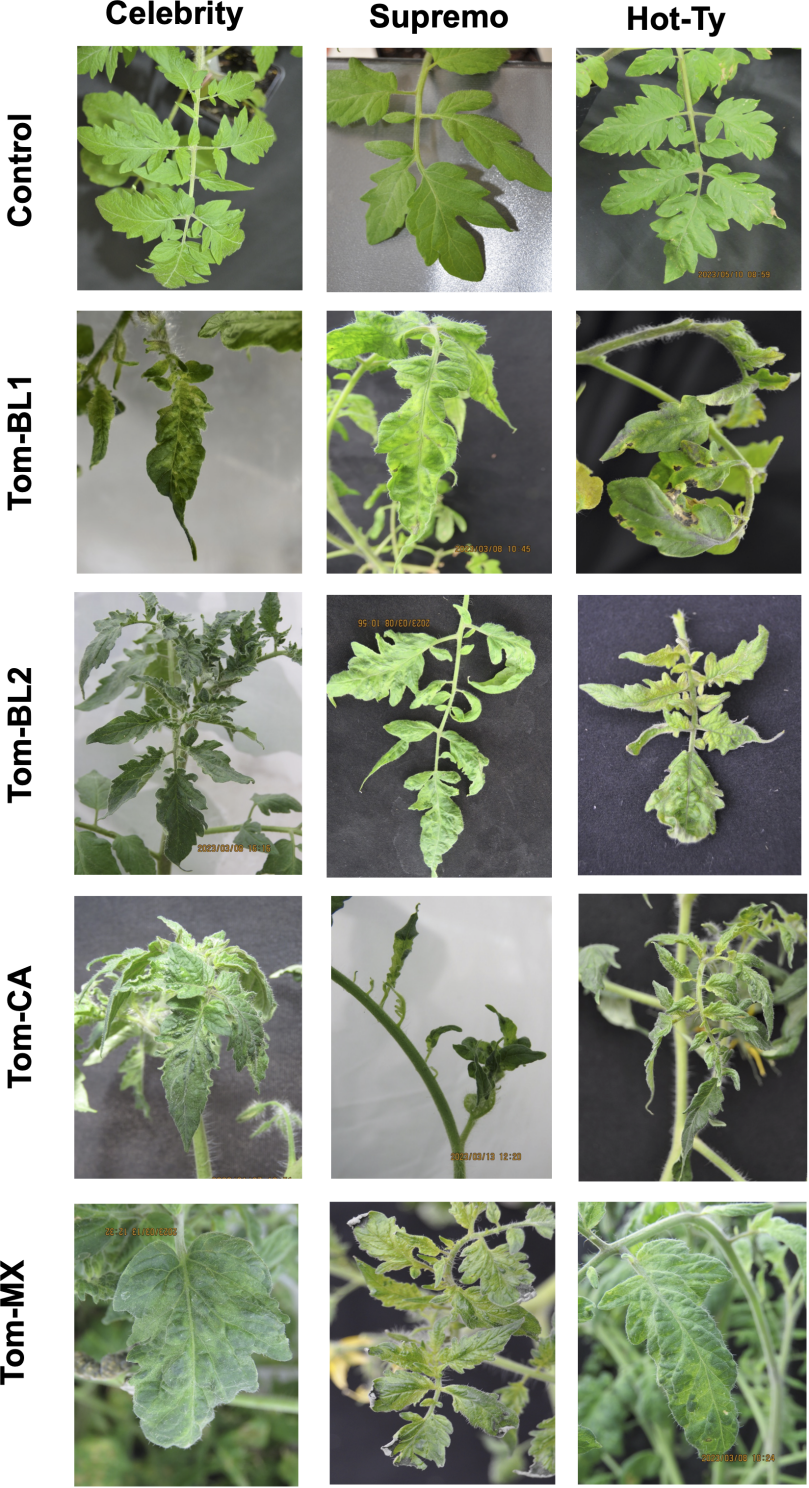
TSW symptoms observed in differentially resistant cultivars infected with sympatric and allopatric strains of TSWV.

TSW symptoms appeared on infected cultivars in a strain-specific manner, while their severity varied across cultivars (**Figure 1**). Across all strains, TSW symptoms were pronounced in susceptible cultivars, followed by both resistant cultivars (**Figure 1**). Across all cultivars, plants infected with sympatric strains (Tom-BL1 and Tom-BL2), produced characteristic symptoms such as chlorotic patches, concentric rings, and necrotic spots on leaves (**Figure 1**). However, in allopatric (Tom-MX and Tom-CA) strain-infected plants, puckering, small-sized leaves, and a mild mosaic of leaves were the most common symptoms. Furthermore, Tom-CA strain induced a shoestring-like leaf symptom which was rarely associated with TSW before (**Figure 1**).

**Figure 2:**
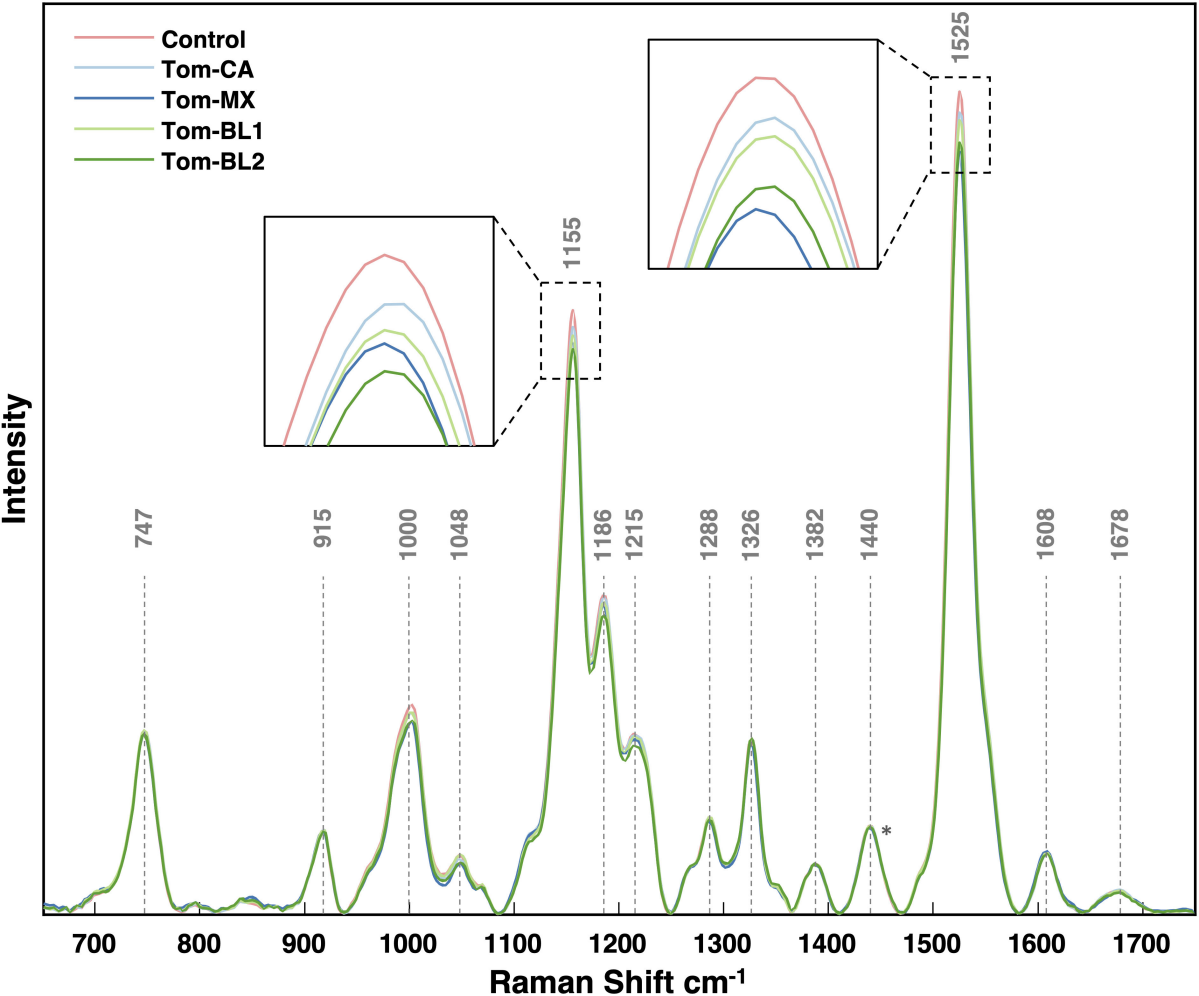
Average Raman spectra collected from each viral strain. Spectra were all normalized at the 1440 peak indicated by an asterisk (*).

Raman spectra obtained from tomato leaves display distinct vibrational bands, corresponding to various biomolecular components. Carbohydrates exhibit bands at 747 and 915 cm^-1^, carotenoids show multiple bands at 1000, 1048, 1155, 1186, 1215, and 1525 cm^-1^, while polyphenols exhibit a band at 1608 cm^-1^ (**Figure 2**, **Table 1**). Additionally, we observed a vibrational band at 1678 cm^-1^, which can be assigned to proteins. Furthermore, CH and CH_2_ vibrations are evident at 1288, 1326, 1382, and 1440 cm^-1^ (**Figure 2**). It is important to highlight that these chemical moieties are widespread across diverse classes of biological molecules. Therefore, 1288, 1326, 1382, and 1440 cm^-1^ vibrations cannot be exclusively attributed to any specific class of biomolecules (**Table 1**).

**Table 1:** Assignments of vibrational bands in the Raman spectra acquired from tomato leaves.

Band (cm^-1^)	Vibrational Mode	Assignment
747	γ(C-O-H) of COOH	Carbohydrates (Synytsya et al., 2003)
915	v(C-O-C) in plane, symmetric	Carbohydrates (Edwards et al., 1997)
1000	in-plane CH3 rocking of polyene	Carotenoids (Adar, 2017)
1048	-C=C-	Carotenoids (Edwards et al., 1997)
1155	-C=C-	Carotenoids (Adar, 2017)
1186	-C=C-	Carotenoids (Adar, 2017)
1215	-C=C-	Carotenoids (Adar, 2017)
1288	δ(C-C-H)	Aliphatics (Yu et al., 2007)
1326	δCH2 bending	Aliphatics (Edwards et al., 1997)
1382	δCH2 bending	Aliphatics (Yu et al., 2007)
1440	δ(CH2) + δ(CH3)	Aliphatics (Yu et al., 2007)
1525	-C=C-	Carotenoids (Adar, 2017)
1608	v(C-C) aromatic ring + σ(CH)	Polyphenols (Agarwal, 2006)
1678	C=O stretching, amide I	Protein (Devitt et al., 2018)

**Figure 3:**
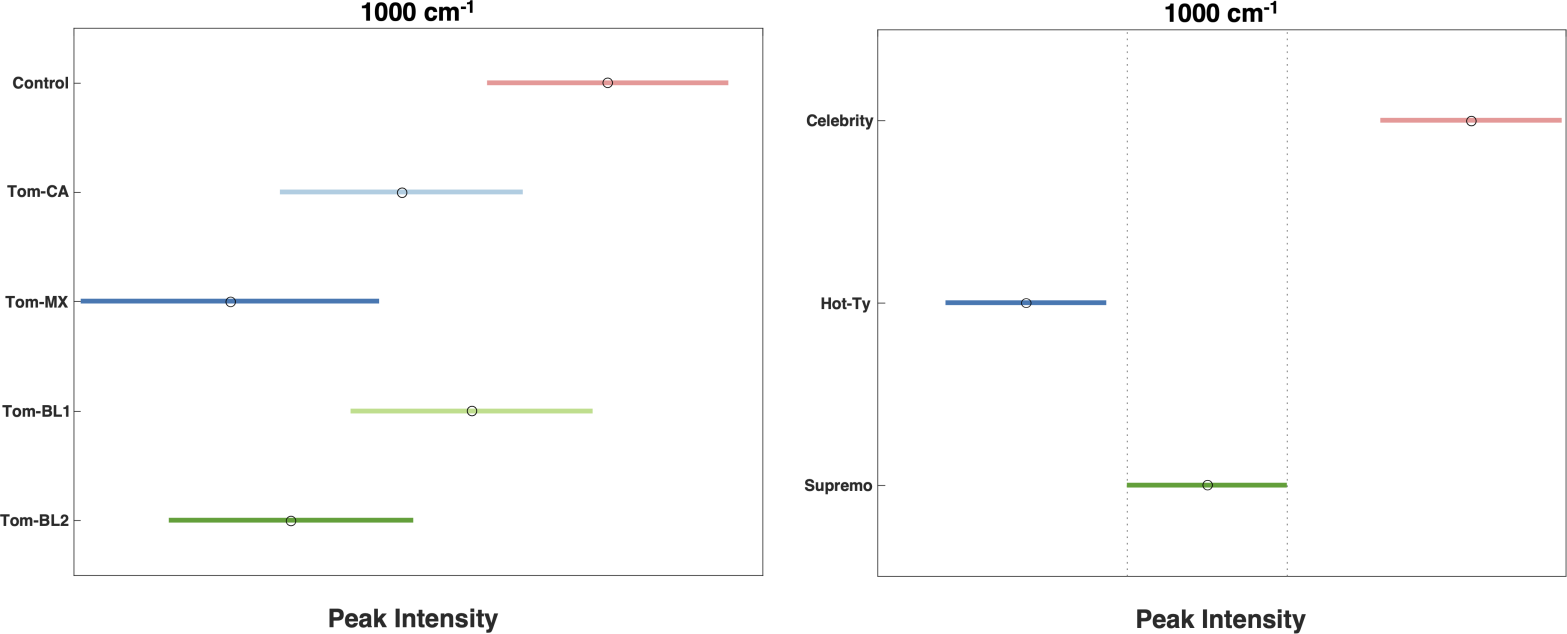
Tukey Test after one-way ANOVA comparing peak intensity by strain (left, p = 5.00 x 10^-4^) and cultivar (right, p = 8.21 x 10^-9^).

By comparing average spectral intensities among TSVW strains, we can monitor biological changes resulting from TSWV. Specifically, a decrease in the peak intensity was observed at 1000, 1048, 1155, 1186, 1215, and 1525 cm^-1^ (**Figure 2**). However, only changes in the intensities of 1000, 1155, and 1525 cm^-1^ bands were statistically significant (**Figure 3**). The Tom-MX and Tom-BL2 strains exerted the most stress to the plants, causing the mentioned decreases in peak intensity. These peaks are all associated with carotenoids, indicating that TSWV infection results in a decreased concentrations of carotenoids in tomato leaves. These findings align well with previously reported results on biotic stress within crops (Higgins et al., 2022). It is worth noting that there was relatively no change in polyphenol content. Earlier research has shown that biotic stress can induce alterations in polyphenol concentration. For instance, in maize, *Colletotrichum graminicola* infection leads to an increase in polyphenols, whereas in wheat, *Diuraphis noxia* infestation results in a decrease (Higgins et al., 2022; Higgins et al., 2023). Viral infections have their own pathogenic mechanisms, distinct from the mechanisms utilized by living pathogens, so this difference could potentially explain why the crops did not experience alterations in polyphenol content.

**Figure 4:**
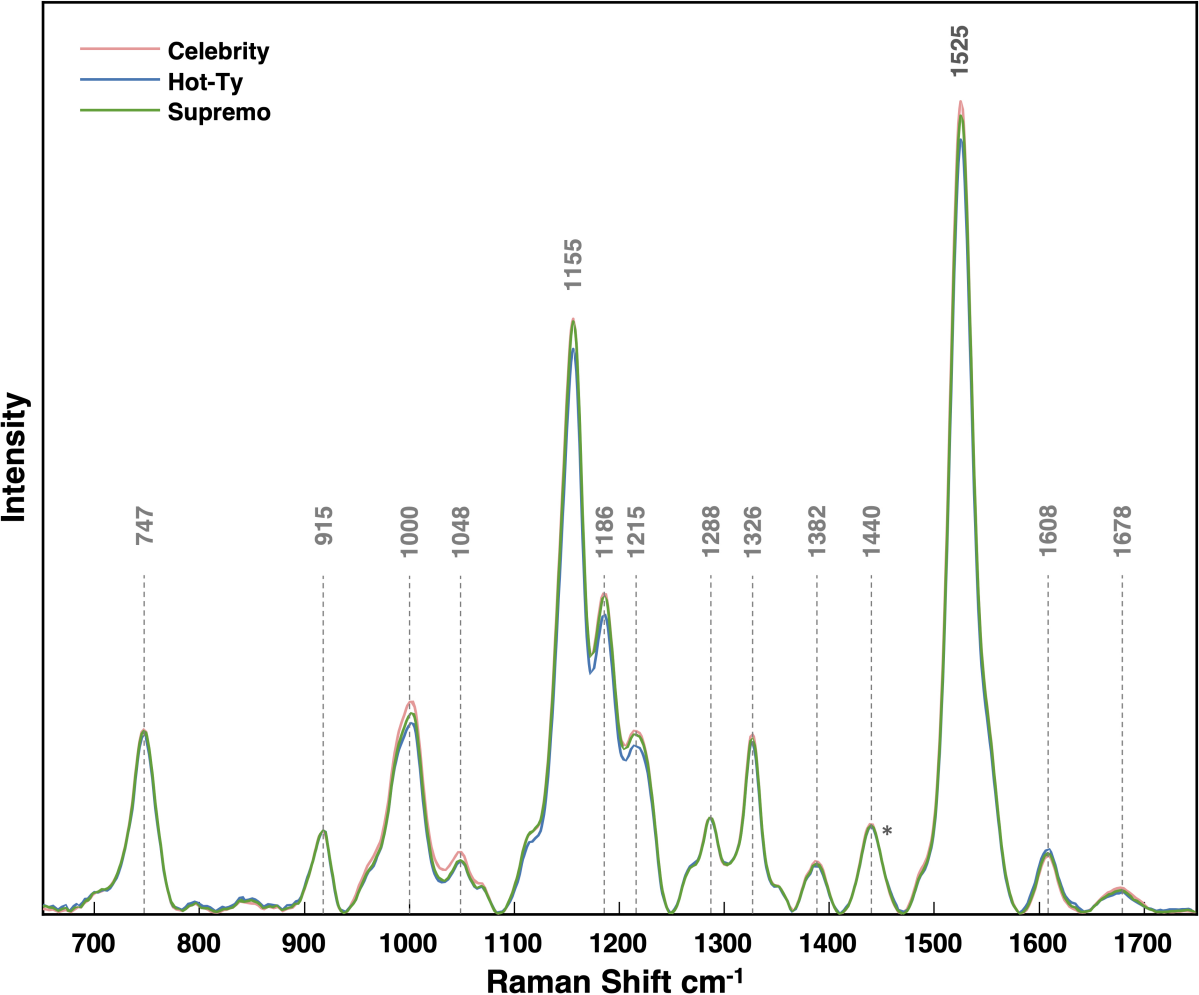
Average Raman spectra collected from each tomato cultivar. Spectra were all normalized at the 1440 peak indicated by an asterisk (*).

When comparing average spectral intensities by cultivar, we found differences in peak intensity at all carotenoid peaks mentioned above (**Figure 4**). Among the three cultivars, Hot-Ty exhibited the lowest average spectral intensity, both visually and statistically, especially in comparison to Celebrity (**Figure 3**). Supremo consistently displayed peak intensities between the values of Celebrity and Hot-Ty. Notably, at all the carotenoid peaks mentioned (1000, 1048, 1155, 1186, 1215, and 1525 cm^-1^), Hot-Ty showed statistically significantly lower intensity values compared to Celebrity. Even though all strains managed to overcome TSWV resistance, these variations in spectral intensities align well with differences in cultivar resistance. Hot-Ty, as a susceptible cultivar, was expected to experience greater stress from the virus, whereas Celebrity and Supremo, as resistant cultivars, were anticipated to fare better.

In addition to examining spectral intensities, we conducted Partial Least Squares-Discriminant Analysis (PLS-DA). This supervised statistical method identifies patterns in data to reveal crucial differences between groups (**Supplementary Figures 2**, **3**). Trained on a labeled dataset, the model classifies individual spectra into specific groups, providing an accuracy percentage that allows us to gauge its predictive capabilities. The model exhibited a high degree of accuracy in predicting both strain and cultivar (**Tables 2**, **3**). Notably, the Control and Tom-MX achieved the most robust prediction rates, whereas some difficulty was encountered in accurately classifying Tom-BL2. The model’s ability to classify cultivars had similar accuracy. While ANOVA and Tukey’s *post-hoc* test results indicated that some groups were not statistically significant from each other, PLS-DA revealed the significance of these variations and their capacity to distinguish different isolates. This predictive power indicates that RS can be used to not only identify viral infection, but to differentiate the infection by cultivar and strain.

**Table 2:** Accuracy of PLS-DA prediction by viral strain.

Strain	Accuracy	Control	Tom-CA	Tom-MX	Tom-BL1	Tom-BL2
Control	90%	81	7	1	1	7
Tom-CA	75%	1	67	0	10	8
Tom-MX	90%	2	0	54	0	3
Tom-BL1	79%	4	11	2	71	10
Tom-BL2	68%	2	4	3	8	60

**Table 3:** Accuracy of PLS-DA prediction by tomato cultivar.

`Cultivar	Accuracy	Celebrity	Hot-Ty	Supremo
`Celebrity	81%	96	16	22
Hot-Ty	76%	11	113	16
Supremo	75%	11	20	112

To validate the outcomes of RS, we performed (RP-HPLC) and analyzed the relative concentrations of lutein (RT = 12.11 min) and chlorophyll (RT = 13.86 min) in the tomato leaves, **Supplementary Figure 4**. Prior research has identified lutein as the predominant carotenoid detected by RS, while alterations in chlorophyll content serve as valuable indicators of plant stress and physiological imbalances (Dou et al., 2021). TSWV suppresses photosynthesis and chloroplast genes, which are essential for lutein and chlorophyll, therefore changes in lutein and chlorophyll content should reflect plant health and align with variations in the Raman spectra (Nachappa et al., 2020).

**Figure 5:**
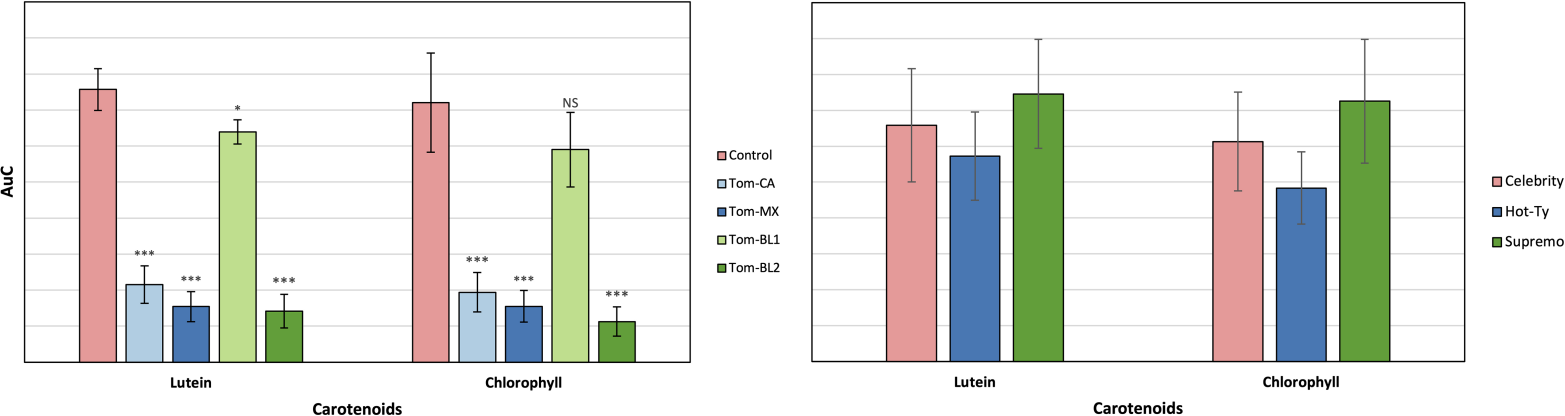
HPLC results by viral strain (left) and tomato cultivar (right). Significance indicate T-test results for the comparison of each strain versus the control: NS is no significance, * is P ≤ 0.05, ** is P ≤ 0.01, and *** is P ≤ 0.001. There was no significance between tomato cultivars.

When grouped by strain, the Tom-MX and Tom-BL2 strains exhibited the lowest concentrations, while the Control had the highest concentration of these molecules (**Figure 5**). These results were consistent with RS data, except for the Tom-CA strain, which showed markedly lower concentration of both lutein and chlorophyll. However, when comparing the HPLC results by cultivar, there was no statistically significant difference between any of the groups. Compared to RS, HPLC inherently contains greater potential error during the analyte extraction, emphasizing the improved dependability of RS in detecting subtle alteration in analyte concentration.

In general, the changes in carotenoid and chlorophyll levels, coupled with the reductions in spectral intensity, align closely with prior findings (Jabeen et al., 2017). The viral defense response of plants has long been associated with the accumulation of reactive oxygen species, with carotenoids playing an important role as antioxidants (Han et al., 2014; Hernández et al., 2016). Carotenoids act as quenchers for singlet oxygen and free radicals in a process that leads to their degradation (Mordi et al., 2020). Furthermore, chlorophyll concentration is well known to decrease during viral infection (González et al., 1997; Kapinga et al., 2009; Mandrile et al., 2022). This provides strong evidence that RS is capable of detecting minute biomolecular changes in content early in TSWV infection. Nevertheless, future work should investigate the limits of TSWV detection and quantify carotenoids changes through gene expression.

## 4 Conclusion

Our findings show that RS can be used for the early TSWV detection in tomato leaves. Significant decreases in carotenoid concentration were noted in several peaks, especially as the Tom-MX and Tom-BL2 isolates produced most severe symptoms on leaves. These results were confirmed by HPLC analysis of lutein and chlorophyll content. Furthermore, we found RS can be coupled with PLSDA to predict the viral strain with around 80% accuracy. These results demonstrate the potential RS has for proactive mitigation of TSWV and for safeguarding food security.

## Data availability statement

The raw data supporting the conclusions of this article will be made available by the authors, without undue reservation.

## Author contributions

IJ: Investigation, Methodology, Validation, Visualization, Writing – original draft, Writing – review & editing. MS: Formal analysis, Investigation, Methodology, Validation, Visualization, Writing – review & editing. SC: Formal analysis, Investigation, Methodology, Validation, Visualization, Writing – review & editing. AR: Formal analysis, Investigation, Methodology, Visualization, Writing – review & editing. KG: Formal analysis, Investigation, Methodology, Validation, Visualization, Writing – review & editing. DK: Conceptualization, Formal analysis, Funding acquisition, Project administration, Resources, Supervision, Writing – original draft, Writing – review & editing.
