# Supplementary material for: Using Raman spectroscopy for early detection of resistance-breaking strains of tomato spotted wilt orthotospovirus in tomatoes
Source: Front Plant Sci. 2024 Jan 3;14:1283399. doi: 10.3389/fpls.2023.1283399 (PMC10791937; doi:10.3389/fpls.2023.1283399)
Supplement: Supplementary file 1 [file DataSheet_1.docx]

Supplementary Material

Using Raman Spectroscopy For Early Detection of Resistance-Breaking Strains of Tomato Spotted Wilt Orthotospovirus in Tomatoes

Isaac D. Juárez, MacKenzi X. Steczkowski, Senthilraja Chinnaiah, Axell Rodriguez, Kiran R. Gadhave^*^, and Dmitry Kurouski^*^

*** Correspondence:** Corresponding Author: kiran.gadhave@tamu.edu, dkurouski@tamu.edu

# Supplementary Figures


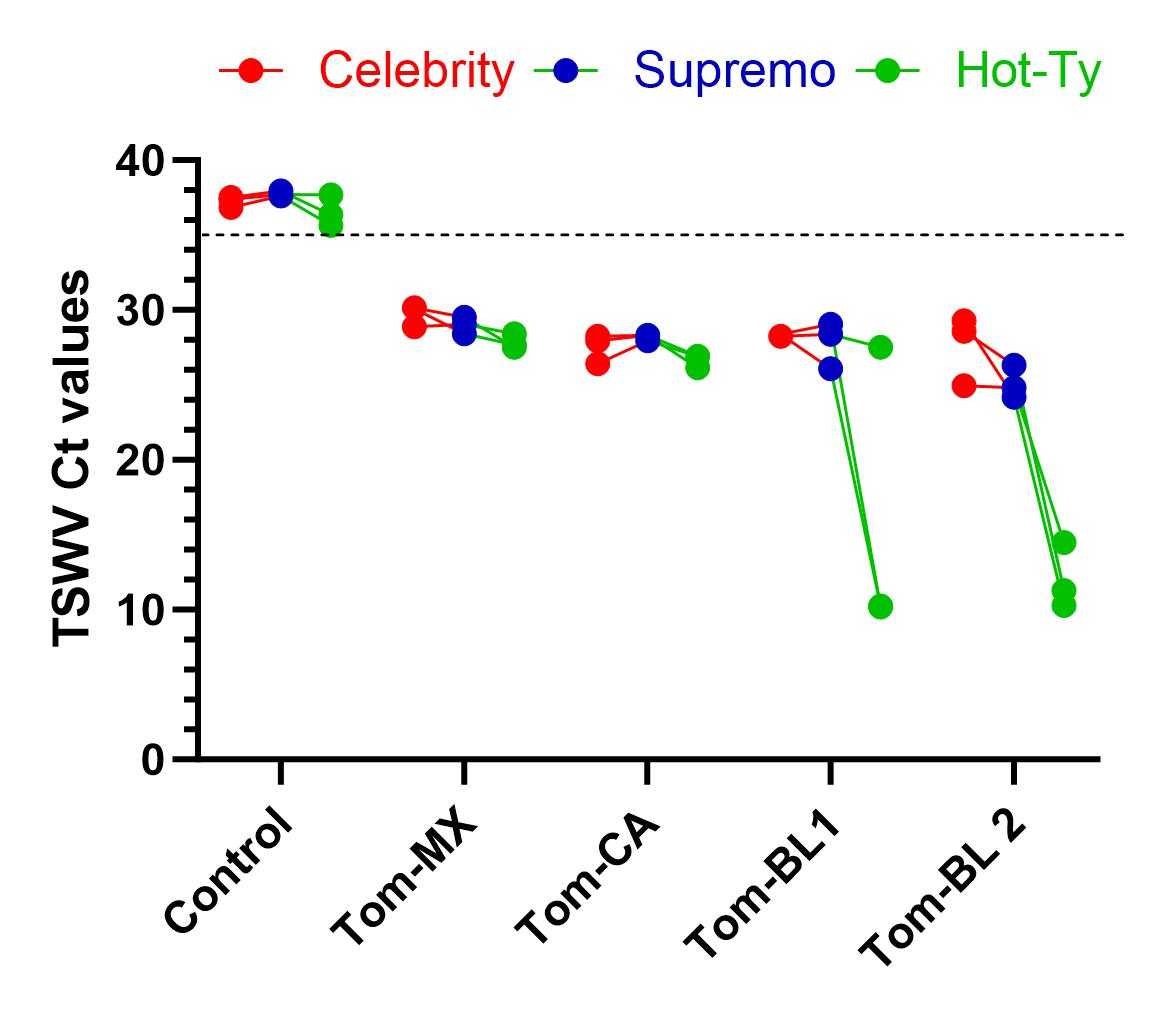


**Supplementary Figure 1.** Quantitative real-time PCR (qPCR) analysis of tomato plants from two TSWV resistant (cv. Celebrity [Red] and Supremo [Blue]) and one susceptible (cv. Hot-Ty [Green]) cultivars infected with four strains of TSWV (Tom-MX, Tom-CA, Tom-BL1 and Tom-BL2) along with the non-infected control. Dotted black line is the Ct cutoff value (Ct ≤ 35) for TSWV-positive samples.


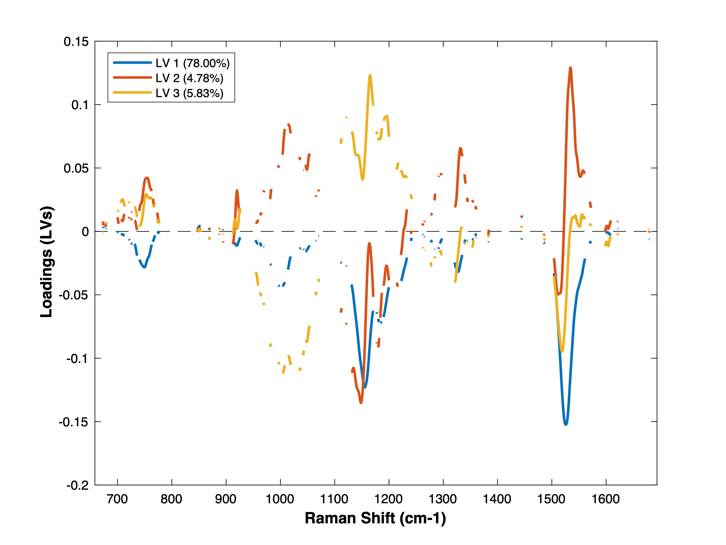


**Supplementary Figure 2.** Loading plot for strain PLS-DA, illustrating the contribution of the most important variables for separation of spectra between groups.

**
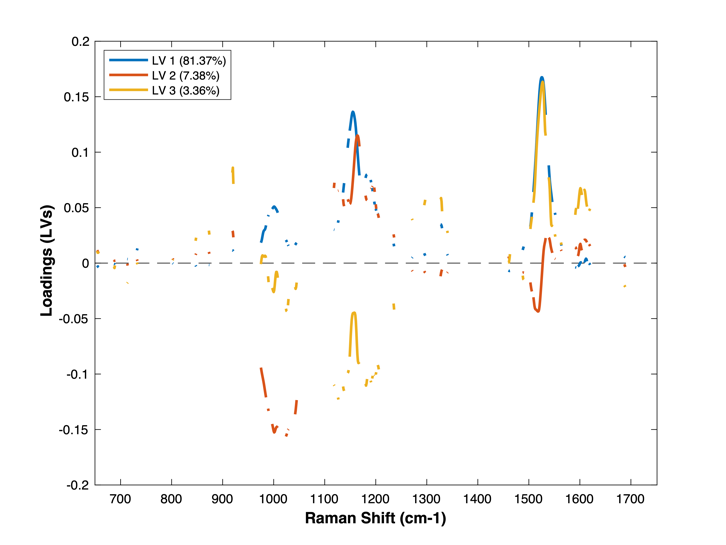
**

**Supplementary Figure 3.** Loading plot for cultivar PLS-DA, illustrating the contribution of the most important variables for separation of spectra between groups.


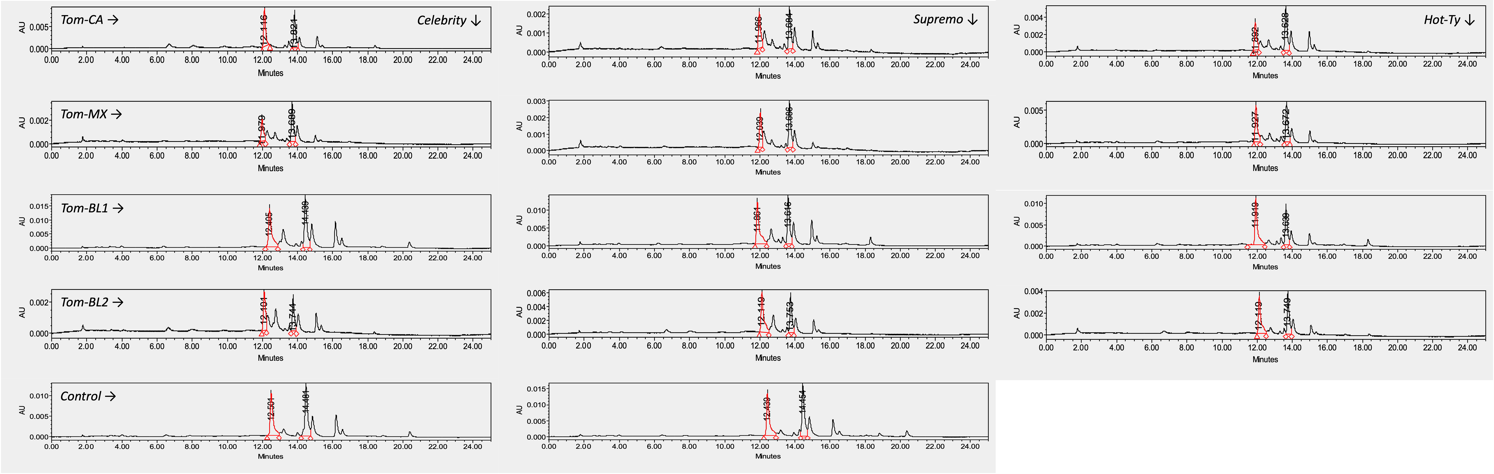


**Supplementary Figure 4.** Results of high-performance liquid chromatograph for each experimental group. The first relevant peak is marked with red.
